# Sensitivity Enhancement of Multiplex Lateral Flow Immunoassays by NIR-II Fluorescence and Thermal Contrast

**DOI:** 10.1021/acs.analchem.5c06734

**Published:** 2026-02-03

**Authors:** Yi-Chi Luo, Yung-Chun Hsieh, Chun-Yang Huang, Yu-Jun Liu, Hsin-Ting Huang, Yan-Chang Chen, Tsung-Yuan Wang, Chong-You Chen, Yang-Hsiang Chan

**Affiliations:** † Department of Applied Chemistry, 34914 National Yang Ming Chiao Tung University, Hsinchu 30010, Taiwan; ‡ Department of Surgery, 63423 National Taiwan University Hospital, Hsinchu Branch, Hsinchu 30010, Taiwan; § Department of Chemistry, 34879 National Taiwan Normal University, Taipei 11677, Taiwan; ∥ Center for Emergent Functional Matter Science, National Yang Ming Chiao Tung University, Hsinchu 30010, Taiwan; ⊥ Department of Medicinal and Applied Chemistry, Kaohsiung Medical University, Kaohsiung 80708, Taiwan

## Abstract

Lateral flow assays (LFAs) are widely used for point-of-care (POC) diagnostics but often suffer from limited sensitivity and specificity compared with laboratory methods. Here, we present a multimodal LFA platform integrating colorimetric, photothermal, and second near-infrared window (NIR-II) fluorescence readouts for enhanced sensitivity and multiplexed detection. Gold nanorods were coupled with bright NIR-II emissive polymer dots to generate plasmon-enhanced fluorescence and efficient photothermal signals within a single probe. As proof-of-concept, carbohydrate antigen 15-3 (CA15-3) and carcinoembryonic antigen (CEA) were selected as target biomarkers for breast cancer screening and broader cancer indication, respectively. Both thermometric and NIR-II fluorescence modes achieved comparable limits of detection for CA15-3 (0.40 and 0.42 U/mL) with a dynamic range of 0–100 U/mL, while CEA was quantified with a detection limit of 0.096 ng/mL. Multiplexed analysis on a single strip allowed simultaneous detection of CA15-3 and CEA with minimal cross-reactivity, and NIR-II fluorescence from test line 2 was intentionally designed to be invisible to the naked eye to avoid interference with rapid CA15-3 screening. Validation with clinical serum samples demonstrated a strong correlation with standard electrochemiluminescence immunoassays. This portable, low-cost platform demonstrates that NIR-II fluorescence and photothermal readouts can be harmonized for sensitive, selective, and multiplexed POC cancer biomarker detection. This universal and signal-amplifying concept can be further adapted to other targets of interest, offering a promising route toward next-generation LFAs.

## Graphical abstract

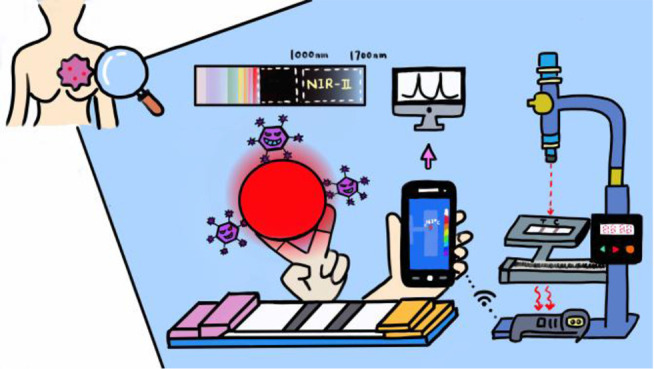

## Introduction

Identifying disease-causing agents timely and accurately worldwide holds transformative potential, which offers a pathway to curb outbreaks and eliminate persistent regional illnesses. The adoption of point-of-care (POC) setting enables rapid diagnosis for urgent care across diverse healthcare scenarios from advanced medical facilities to resource-limited environments.^1–3^ During the last 20 years, point-of-care diagnostic technologies have advanced significantly to address these needs. In particular, lateral flow assays (LFAs) have gained prominence due to their affordability, ease of use, and broad applicability to the detection of serum analytes, viral pathogens, toxins, bacteria, and proteins.^4–6^ Their importance was further highlighted during the SARS-CoV-2 outbreak beginning in 2019 as LFAs further gained prominence as vital diagnostic tools, emphasizing their indispensable role in pandemic response strategies.

Unfortunately, traditionally built commercial LFAs face notable drawbacks when analyzing complex samples, including reduced analytical performance compared to laboratory-based methods.^6,7^ Their limited sensitivity and selectivity frequently hinder reliable quantification, which is critical for guiding targeted treatments. Concurrently, advancements in multiplexed LFA development aim to address these gaps by enabling simultaneous detection of multiple biomarkers.^5,8,9^ Such innovations promise cost efficiency, minimized sample requirements, and faster differentiation of diseases with common symptoms. Despite these benefits, technical hurdles remain, including unintended cross-interactions among detection analytes, spatial limitations on test strips, and the need for rigorous clinical validation that complicates widespread implementation.^8,10^

Aiming to attain multiplexed detection capability with enhanced sensitivity and selectivity in LFAs, recent efforts have increasingly concentrated on incorporating more than one signal readout mechanism within a single lateral flow assay, leading to the emergence of dual- and trimodal LFA platforms.^11,12^ A common strategy pairs visual color readouts with fluorescence-based measurements, allowing intuitive interpretation alongside precise quantification and typically improving detection limits by several hundred- to thousand-fold relative to conventional single-readout LFAs.^8^ Building on this approach, recent progress has produced multimodal LFAs that integrate diverse detection methods, such as colorimetric, fluorescent, magnetic, photothermal, electrochemical, and scattering signal modalities.^8^ While promising, many of the aforementioned approaches rely on advanced instrumentation such as spectrometers, which compromise their suitability for POC applications. Among these new platforms, fluorescent^4^ and thermometric^13–16^ LFAs simply require add-on readers (e.g., smartphones or infrared cameras) based on traditional colorimetric LFA systems to significantly enhance detection sensitivity. However, a fundamental challenge arises because fluorescent and photothermal mechanisms are diametrically opposed, as photothermal signals depend on nonradiative energy conversion while fluorescence requires efficient radiative decay. This conflict highlights the need for innovative multimodal readout systems that harmonize fluorescent and thermometric signals, which could unlock improved performance in multiplexed LFAs.

Achieving simultaneous fluorescent and thermometric signals requires the strategic integration of complementary reporters. In terms of fluorescence, NIR-II (1000–1700 nm) emissive probes are ideal candidates due to their reduced light scattering, minimal tissue autofluorescence, and inherent photothermal conversion capabilities within the NIR-II window.^17–25^ For thermal contrast agents, gold-based nanostructures are prioritized, owing to their chemical inertness, strong surface plasmon resonance absorption, and efficient photothermal energy conversion under light irradiation. To prove our proposed concept, we synthesized a highly bright NIR-II fluorescent polymer and engineered it into water-dispersible polymer dots (Pdots). In parallel, we prepared Au nanorods (NRs) as dual-functional agents, serving as both photothermal contrast reporters and colorimetric indicators via their surface plasmon resonance properties. By coating Pdots onto Au nanorods, we constructed a dual-modal LFA system capable of qualitative and quantitative multiplexed detection. As a proof-of-concept, this proposed system was employed for the simultaneous detection of two clinically relevant prognostic markers associated with breast cancer: carbohydrate antigen 15-3 (CA15-3) and carcinoembryonic antigen (CEA). The CA15-3 signal is used for rapid screening of breast cancer, while the CEA serves as an indicator of the potential presence of other cancers. Notably, the NIR-II fluorescence-based detection limit for CA15-3 reached 0.42 U/mL, which is comparable to that achieved via thermal signaling (0.40 U/mL). This demonstrates the superiority of NIR-II fluorescence over visible-range emission, as also validated in our study. Besides, the values generated using this method closely matched those obtained from ELISA which confirms the robustness and quantitative validity of the proposed LFA system. This advanced assay design therefore provides a promising route toward fast and accurate point-of-care testing of cancer-related biomarkers.

## Experimental Section

### Preparation of Au Nanorods

Gold nanorods were synthesized by using a seed-mediated growth strategy. Briefly, the seed dispersion was first prepared by combining an aqueous solution of HAuCl_4_·3H_2_O (25 μL, 10 mM) with CTAB (1 mL, 0.1 M) in a 1.7 mL microcentrifuge tube, followed by thorough mixing. A freshly prepared ice-cold NaBH_4_ solution (55 μL, 0.01 M) was then rapidly introduced, and the mixture was immediately agitated for 2 min. The solution color transitioned from yellow to pale brown, after which it was aged in a 30 °C water bath for 2 h to obtain the gold seed solution. For nanorod growth, HAuCl_4_·3H_2_O (500 μL, 10 mM), CTAB (10 mL, 0.1 M), AgNO_3_ (37 μL, 0.01 M), HCl (250 μL, 1 N), and ascorbic acid (80 μL, 0.1 M) were sequentially added to a 15 mL centrifuge tube with gentle mixing. Upon reduction, the solution became colorless. Subsequently, 25 μL of the seed solution was introduced, and the reaction mixture was maintained at 30 °C for 6 h. The growth process was quenched by adding NaOH (250 μL, 1 M), resulting in a blue-green dispersion. Formation of gold nanorods was confirmed by UV–vis spectroscopy, showing a dominant longitudinal plasmon band near 650 nm and a weaker transverse band around 525 nm.

### Preparation of Pdots

BDT-TTQ, carboxymethyl–PEG–DSPE (CM-DSPE, *M*_W_ = 2000), thiol-functionalized polystyrene (PS-SH, *M*_n_ ≈ 25 kDa), and a cumene-terminated styrenic polymer (PSMA, *M*_n_ ≈ 1.9 kDa; styrene content 75%) were each prepared as separate stock solutions in THF at a concentration of 1 mg/mL. To assemble the nanoparticle formulation, THF (2.5 mL) was first introduced into a 20 mL glass vial, followed by the sequential addition of BDT-TTQ (150 μL), PS-SH (0–10 μL), CM-DSPE (20–30 μL), and PSMA (5 μL). The mixture was homogenized to yield a uniform organic phase. This solution was then rapidly transferred to 5 mL of deionized water contained in a separate vial under continuous ultrasonication. Solvent removal was achieved by heating the dispersion at 70 °C under a nitrogen atmosphere for approximately 20 min until THF was fully eliminated, leaving an aqueous suspension with a final volume of ∼4 mL. Upon cooling to room temperature, the dispersion was passed through a 0.22 μm cellulose acetate syringe filter to remove oversized particles and aggregates.

### Fabrication of AuNR@Pdot Nanohybrid Probes

A suspension containing BDT-TTQ Pdots (2 mL) and Au nanorods (1 mL) was combined with 0.75 mL of 1 M HEPES buffer and gently agitated for 50 min. The mixture was then aliquoted into centrifuge tubes and spun at 7000 rpm for 10 min. Following centrifugation, the clear supernatant was removed, and the collected precipitate was redispersed in 1 mL of deionized water. The resulting AuNR@Pdot dispersion was stable when stored at 4 °C for several weeks.

### Surface Modification of Boronic Acid on AuNR@Pdot Nanohybrids

AuNR@Pdot nanohybrids (1.0 mL) were combined with HEPES buffer (20 μL, 1 M), freshly prepared EDC solution (20 μL, 5 mg/mL), NHS solution (5 μL, 5 mg/mL), polyethylene glycol (20 μL, 5 wt %, *M*_n_ = 3350), and 4-aminomethylphenylboronic acid hydrochloride (AMPB, 25 μL, 1 mg/mL). The reaction mixture was gently stirred for 3.5 h to allow the conjugation to proceed. Following incubation, the suspension was transferred to centrifuge tubes and collected by centrifugation at 7000 rpm for 8 min. After centrifugation, the supernatant was removed, and 0.2 mL of 10 mM HEPES was introduced to resuspend the pellet. The resulting AMPB-modified AuNR@Pdot solution can be stored at 4 °C for 3–4 weeks. For long-term storage, the AuNR@Pdot could be further purified through a size exclusion chromatography resin column (Sephacryl S-300 HR) using 5% (w/w) PEG solution as the eluent.

### Antibody Conjugation of Boronic Acid-Functionalized AuNR@Pdot

To functionalize CA15-3 antibodies onto the surface of AuNR@Pdot, 2 μL of CA15-3 antibodies (1 mg/mL, product code: 136549, United States Biological Corporation) and 200 μL of boronic acid-functionalized AuNR@Pdot were mixed and incubated in a water bath at 37 °C for 1.5 h. After the reaction, the detection probes were ready to use or could be stored at 4 °C. For the conjugation with CEA antibodies, 1.6 μL of CEA antibodies (1 mg/mL, product code: 7882, United States Biological Corporation) was used instead.

### Measurement of Thermal Signal by IR Camera-Connected Smartphone on Lateral Flow Strip

1) Remove the sample pad and absorbent pad from the test strip after sample detection and allow the nitrocellulose membrane to dry completely. 2) Use a utility knife to cut the nitrocellulose membrane from the backing plate. 3) Place the test strip on the thermal detection platform and turn on the portable/miniature 650 nm laser pointer (can be powered by USB-C) as the excitation light. Use the control buttons to move the test strip horizontally and sequentially measure the temperature and image of 16 points with 2 s/point of retention time from left to right on the nitrocellulose membrane using a portable thermal camera (FLIR ONE Edge Pro, Teledyne FLIR). The measurement data are transmitted via Wi-Fi to a smartphone for recording experimental results and data. The entire procedure is demonstrated in Video S1. For each analyte concentration, a minimum of five independent measurements was performed to calculate the corresponding standard deviations.

## Results and Discussion

Aiming at the design of a multimodal LFA that integrates colorimetric, fluorescent, and/or photothermal signals for the concurrent detection of CA15-3 and CEA in clinical specimens, we designed a hybrid probe that can generate all three signal modalities. Also, we intend to compare the sensitivities of the fluorescent and thermometric modes, which are among the most sensitive detection strategies currently used in LFAs. Strategically, gold nanorods were employed as photothermal agents and integrated with NIR-II emissive Pdots within a single probe to enable dual-signal generation for sensitivity comparison. In clinical applications, the colorimetric signal allows for rapid screening of CA15-3 for breast cancer, while subsequent detection of CEA may indicate the presence of other types of cancer.

### Setup of Portable Thermometric LFA Platform

To meet the requirements of POC applications, we developed a portable thermometric reader, as illustrated in Figure 1. The setup includes a 3D-printed plastic sample holder mounted on a stainless-steel lab stand lift table, driven by an Arduino-controlled stepper motor (DM542S/CT-28-0602-100, HaiJei-tech, China). A 650 nm portable laser (5 V, 200 mW) was secured on a camera slider for vertical movement to allow vertical adjustment for precise laser focusing on the test strip. The sample can be moved horizontally across the laser spot at a constant scanning speed via the stepper motor. A rechargeable thermal camera (FLIR ONE Edge Pro) is positioned beneath the test strip to capture thermal signals with data transmitted wirelessly to a smartphone (Android or iOS) using the FLIR ONE app. The entire system is compact, mobile, and costs under USD 1,000, making it ideal for POC.

**Figure 1:**
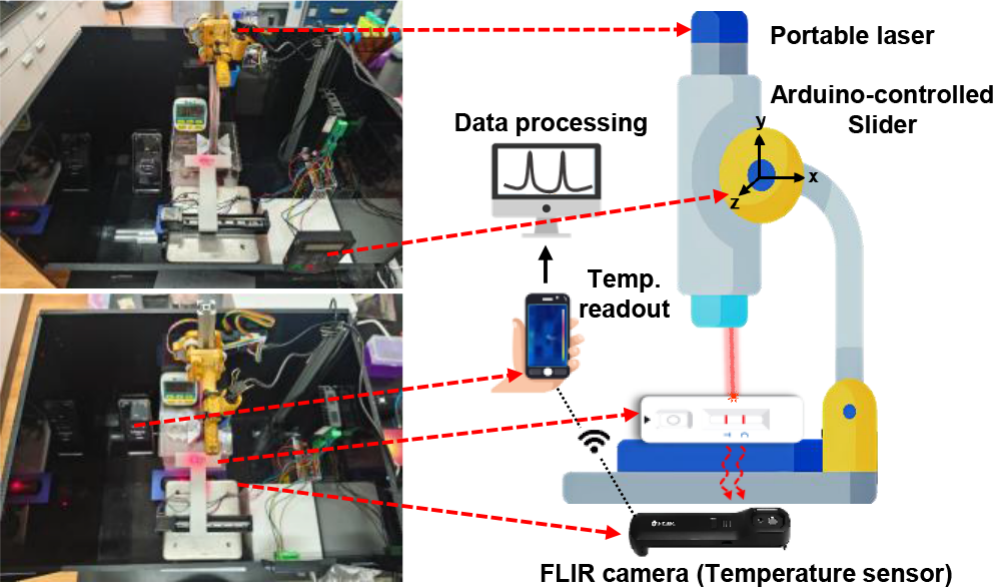
Setup of a portable thermometric LFA platform. The left two photographs represent a customized thermometric LFA reader and the right figure shows its cartoonized layout. Major components include an Arduino-controlled motorized slider, a 200 mW 650 nm laser, an IR camera, and a smartphone.

### Preparation of AuNR@Pdot Dual-Signal Probes

Attempting to design a hybrid nanoprobe with both photothermal and NIR-II fluorescence properties, we took advantage of bright NIR-II emissive Pdots and the efficient photothermal conversion of AuNRs. To select a good pair of AuNRs and Pdots, we first synthesized AuNRs with different aspect ratios to tune their surface plasmon resonance at distinct wavelengths. As shown in Figure 2A, three types of AuNRs exhibited absorption peaks at around 530 nm (solid red line), 650 nm (solid blue line), and 850 nm (solid purple line), respectively. Next, we synthesized a highly NIR-II fluorescent polymer, BDT-TTQ, and coated it onto Pdots to form the hybrid structure illustrated in Scheme 1A. The resulting AuNR@Pdots nanohybrids (dashed lines in Figure 2A) retained similar absorption profiles to their bare AuNR counterparts, indicating that the photothermal properties of AuNRs were preserved after Pdot encapsulation. We then evaluated the fluorescence intensities of these hybrids and found that those incorporating AuNRs with a 650 nm surface plasmon resonance peak exhibited the highest NIR-II emission (approximately 1.5-fold stronger than bare Pdots; ) in both solution and test strip formats (Figure 2B). These findings align with previous reports,^26,27^ emphasizing the importance of selecting an optimal plasmonic absorption wavelength for fluorescence enhancement. Thus, we selected 650 nm AuNRs for further experiments. Dynamic light scattering (DLS) and transmission electron microscope (TEM) data confirmed successful Pdot coating on the AuNR surface (Figure 2C–H). The electrostatic interaction between negatively charged Pdots (ζ = −14.1 mV) and positively charged AuNRs (ζ = +33.4 mV) led to the formation of negatively charged AuNR@Pdots hybrids (ζ = −21.5 mV) (Figure 2I). The negative surface charge is advantageous for lateral flow applications, because nitrocellulose membranes typically repel positively charged probes, reducing nonspecific binding. Upon conjugation with AMPB chelators (Scheme 1A,B), the zeta potential remained stable (ζ = −22.4 mV), indicating the good colloidal stability of AuNR@Pdots. Notably, in this study, the AMPB chelator was employed as a linker between the Pdot surface (boronic acids) and antibodies (carbohydrates in the Fc region) rather than directly conjugating antibodies to the Pdots. Direct coupling of antibody amine groups (Fab region) to the carboxyl groups on Pdots often results in improper (head-on) orientation, which can significantly reduce both the sensitivity and selectivity toward target antigens.^28^

**Figure 2:**
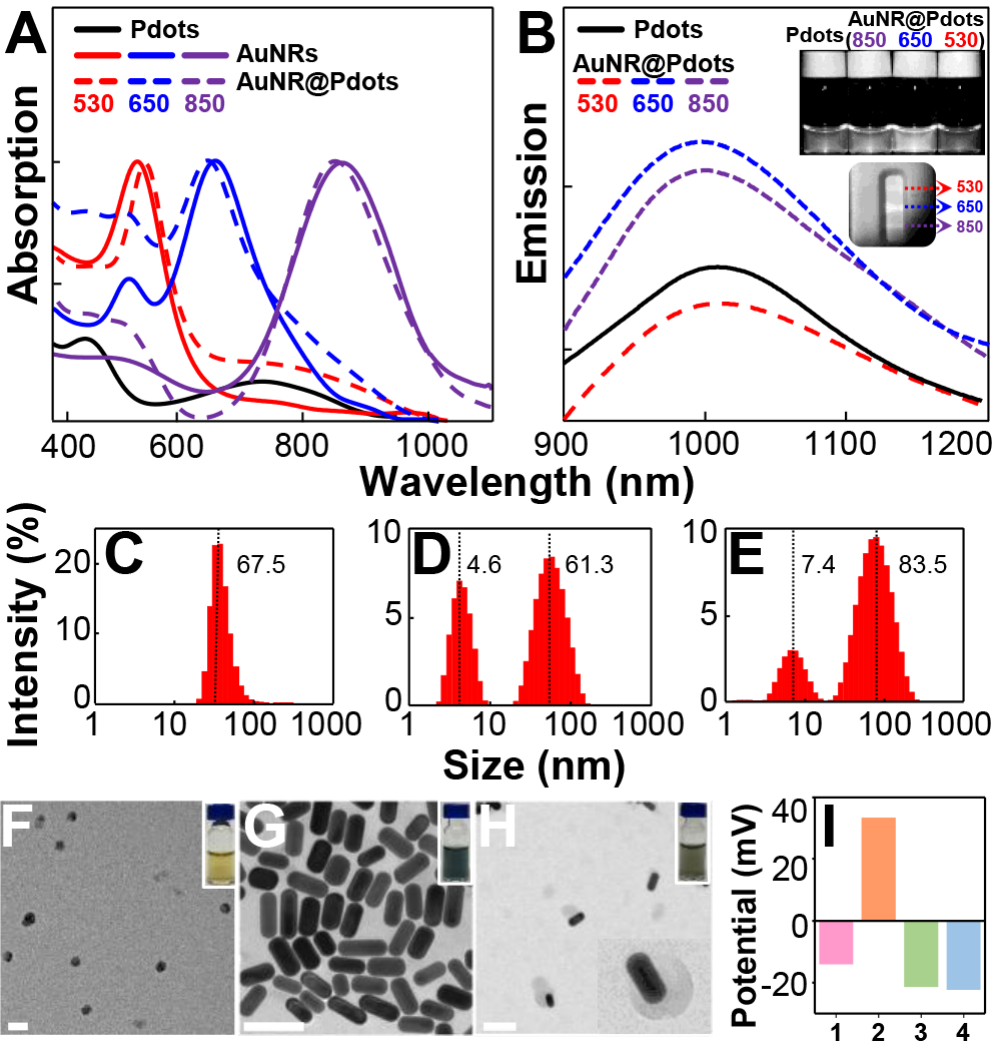
(A) UV–vis absorption spectra of Pdots (black line), AuNRs (solid red, blue, and purple lines), and AuNR@Pdots (corresponding dashed lines) in water. (B) Fluorescence emission spectra of Pdots (solid black line) and AuNR@Pdots (dashed red, blue, and purple lines) in aqueous solution. The upper-right inset displays photographs of the samples under 633 nm laser illumination. The middle-right inset shows the lines using three types of AuNR@Pdots in a test strip under 633 nm laser excitation. Hydrodynamic diameters of (C) bare Pdots, (D) AuNRs, and (E) AuNR@Pdots; their corresponding TEM images are shown in (F), (G), and (H), respectively. The upper-right insets in (F–H) display the photographs of each solution under room light, and the bottom-right inset in (H) represents the enlarged TEM image of a single AuNR@Pdot nanohybrid. The scale bars are 100 nm. (I) Zeta potentials of bare Pdots (No. 1), AuNRs (No. 2), AuNR@Pdots (No. 3), and AMPB-functionalized AuNR@Pdots (No. 4).

**Scheme 1:**
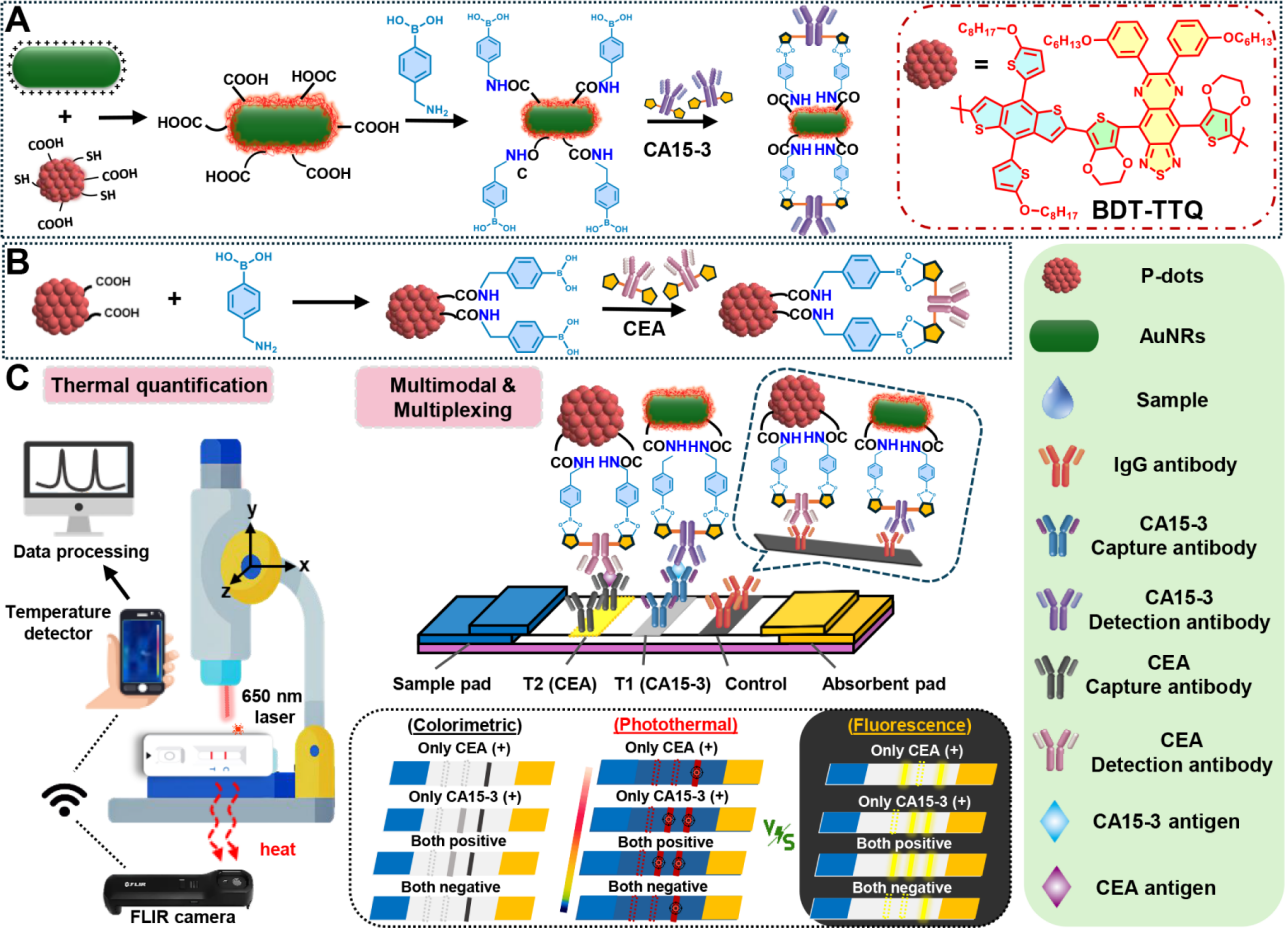
Schematic Illustrating the Design of AuNR@Pdots with NIR-II Emission and Thermometric Modes for Test Strip^a^

a AuNRs (A) and NIR-II emissive Pdots (B) were first prepared separately. For the preparation of AuNR@Pdots, negatively charged Pdots were coated onto the positively charged AuNRs, followed by the conjugation of AMPB. The detection antibodies of CA15-3 and CEA were bound onto the surface of AuNR@Pdots and Pdots, respectively. (C) A portable thermometric LFA platform was used to measure the thermometric signals of AuNR@Pdots. The test strip was fabricated with one control line modified by an IgG secondary antibody, test line 1 modified by a CA15-3 capture antibody, and test line 2 modified by a CEA capture antibody. Three modalities, including colorimetric, photothermal, and fluorescent signals, can be reported simultaneously. The color shade from AuNRs can be used for quick screening, while both the thermometric and NIR-II fluorescent readouts allow for quantitative measurement of antigen levels.

### Detection Mechanism of AuNr@Pdot-Based LFA

Thermometric and fluorescent modalities represent two of the most effective platforms for enhancing sensitivity in LFAs, while maintaining their inherent portability. By coupling NIR-II fluorescent Pdots with AuNRs, we achieved successful integration of plasmon-enhanced fluorescence and photothermal signaling. As a proof-of-concept, CA15-3, a breast cancer biomarker, was selected as the target analyte. In our test strip design (Scheme 1C), the control line and two test lines were functionalized with secondary IgG antibodies, CA15-3 capture antibodies, and CEA antibodies, respectively. The CA15-3 test line is intended for rapid breast cancer screening, while the CEA test line serves as an optional indicator for other malignancies. From a clinico-pathological perspective, serum CA15-3 levels are closely associated with malignant breast cancer and often correlate with tumor stage.^29^ In contrast, CEA is a nonspecific biomarker elevated in various cancers,^30^ including but not limited to breast cancer. Therefore, CEA levels can vary widely and do not reliably distinguish between cancer types. In an ideal use case, visually positive test line 1 (CA15-3) can be employed for first-round breast cancer screening and monitoring, regardless of the CEA test line result. Test line 2 (CEA), which is intentionally designed to be invisible to the naked eye, helps avoid visual interference and serves to evaluate the likelihood of other cancers when test line 1 is negative. Both test lines also support quantitative analysis of their respective biomarkers. We further compared the sensitivity performances of photothermal and NIR-II fluorescence readouts. This thermometric–fluorescent–colorimetric multimode LFA represents a promising strategy for enhancing assay sensitivity and enabling multiplexed detection.

### Selectivity Assessment of NIR-II Pdot-Based LFA

We first assessed the specificity of the LFA platform using several common tumor biomarkers. In the first set of experiments, each strip contained only a control line and test line 1 to evaluate the selectivity of the CA15-3-conjugated AuNR@Pdots. As shown in Figure 3A, the probes displayed excellent specificity for CA15-3, with minimal nonspecific adsorption to other interfering antigens, including AFP, CEA, CA125, and CYFRA 21-2. The colorimetric mode (upper panel) was less distinguishable than the NIR-II fluorescence mode (lower panel), suggesting that NIR-II emission is well suited for quantitative analysis, whereas the probe’s color shade enables rapid naked-eye screening. In NIR-II mode, the fluorescence intensity at test line 1 for CA15-3 was more than 17-fold higher than those for the other antigens (blue columns, Figure 3C). In photothermal mode, the thermal contrast ratio between the test and control lines for CA15-3 was over 18-fold higher than that for the other reagents (pink columns, Figure 3C). These results indicate that plasmon-enhanced NIR-II fluorescence and photothermal readouts offer comparable sensitivity, while NIR-II fluorescence is operationally more convenient. In the second set of experiments, strips were fabricated with a control line and test line 2 to examine the selectivity of CEA-functionalized Pdots. As shown in Figure 3B, the probes exhibited exceptional selectivity for CEA over other tumor markers, with test-to-control line emission ratios exceeding 15-fold for CEA compared with other analytes (orange columns, Figure 3C). These findings show that even bare NIR-II fluorescence, without plasmonic enhancement, can match the selectivity of thermometric signals. We further confirmed the higher sensitivity of NIR-II emission compared with traditional visible emission (), attributed to the exceptionally low background signals within the NIR-II spectral range, which improves the signal-to-background ratio. Finally, reaction-time analysis revealed that both fluorescence and temperature signals reached equilibrium within 15 min (Figure 3D), meeting the requirements for point-of-care applications.

**Figure 3:**
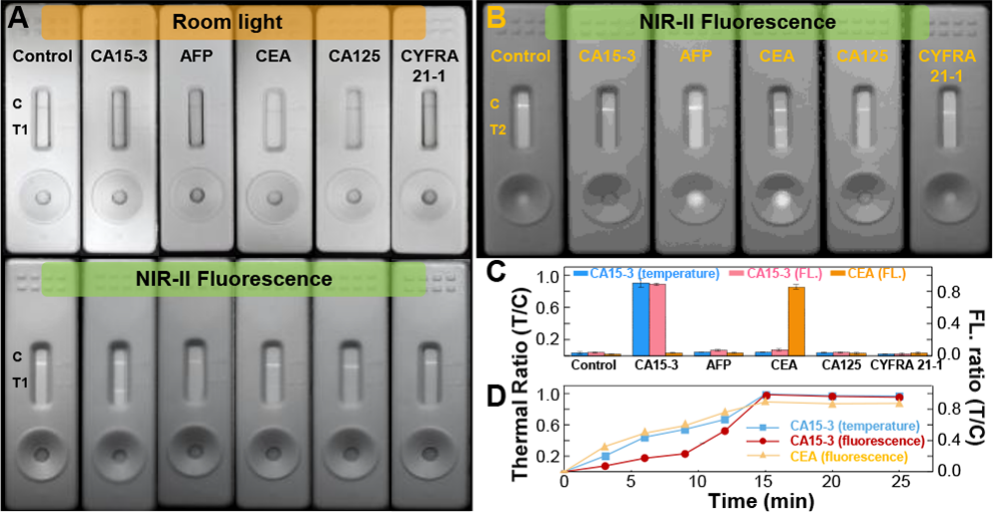
Selectivity evaluation of NIR-II Pdot-based test strips (CA15-3: 100 U/mL; AFP: 30 ng/mL; CEA: 7 ng/mL; CA125: 50 ng/mL; CYFRA21-1: 5 ng/mL). (A) Test strips functionalized with CA15-3 targeting AuNR@Pdot probes after exposure to various antigens. Each strip contains a control line (IgG) and test line 1 (CA15-3). The upper panel shows photographs under ambient light, and the lower panel presents the corresponding NIR-II fluorescence images acquired under 650 nm flashlight illumination by an Olympus camera fitted with an 830 nm long-pass emission filter. (B) NIR-II fluorescence images of test strips functionalized with CEA-conjugated Pdots after reaction with different analytes. Each strip contains a control line (IgG) and test line 2 (CEA). (C) Bar graph summarizing the test-to-control (T/C) values after 15 min of reaction. (D) Ratios of T/C temperature increase and/or fluorescence intensity for CA15-3 test strips in (A) and CEA test strips in (B) over various reaction times.

### Comparison of Detection Sensitivity: Thermal Contrast versus NIR-II Fluorescence

We compared the detection sensitivity of thermal contrast and NIR-II fluorescence, two of the most sensitive LFA modalities. AuNR@Pdot nanohybrids functionalized with CA15-3 detection antibodies were used as probes on test strips containing a control line (IgG) and a test line 1 (CA15-3 capture antibody) at varying CA15-3 concentrations. As shown in Figure 4A, the test lines became visibly darker with an increase in CA15-3 levels. For thermometric readout, a 650 nm laser scanned the strip from the sample pad, across the test and control regions, and into the absorbent pad. Temperatures at 16 points (1 mm intervals) were recorded with a thermal camera, and the temperature increases were calculated as summarized in Figure 4B (0–30 U/mL) and Figure 4K (50–130 U/mL). Thermal images for each scanning point at different CA15-3 levels (0–130 U/mL) are shown in Figure 4C–J, with the first image of each panel representing a background point. The laser retention time was kept under 2 s to prevent damage to the nitrocellulose membrane. Both thermometric and fluorescence modalities exhibited a dynamic range spanning 0–100 U/mL (Figure 4L–N), with detection limits determined to be 0.40 and 0.42 U/mL, respectively, indicating comparable sensitivity and linearity. Thermal contrast is straightforward to read but requires skilled operation of the instrumentation, whereas NIR-II fluorescence images can be rapidly acquired using a customized digital camera, though precise quantification may require specialized software. Each method offers distinct advantages, and our results demonstrate that their sensitivity and specificity are comparable. Ultimately, the choice between the two depends on the specific requirements of the point-of-care setting.

**Figure 4:**
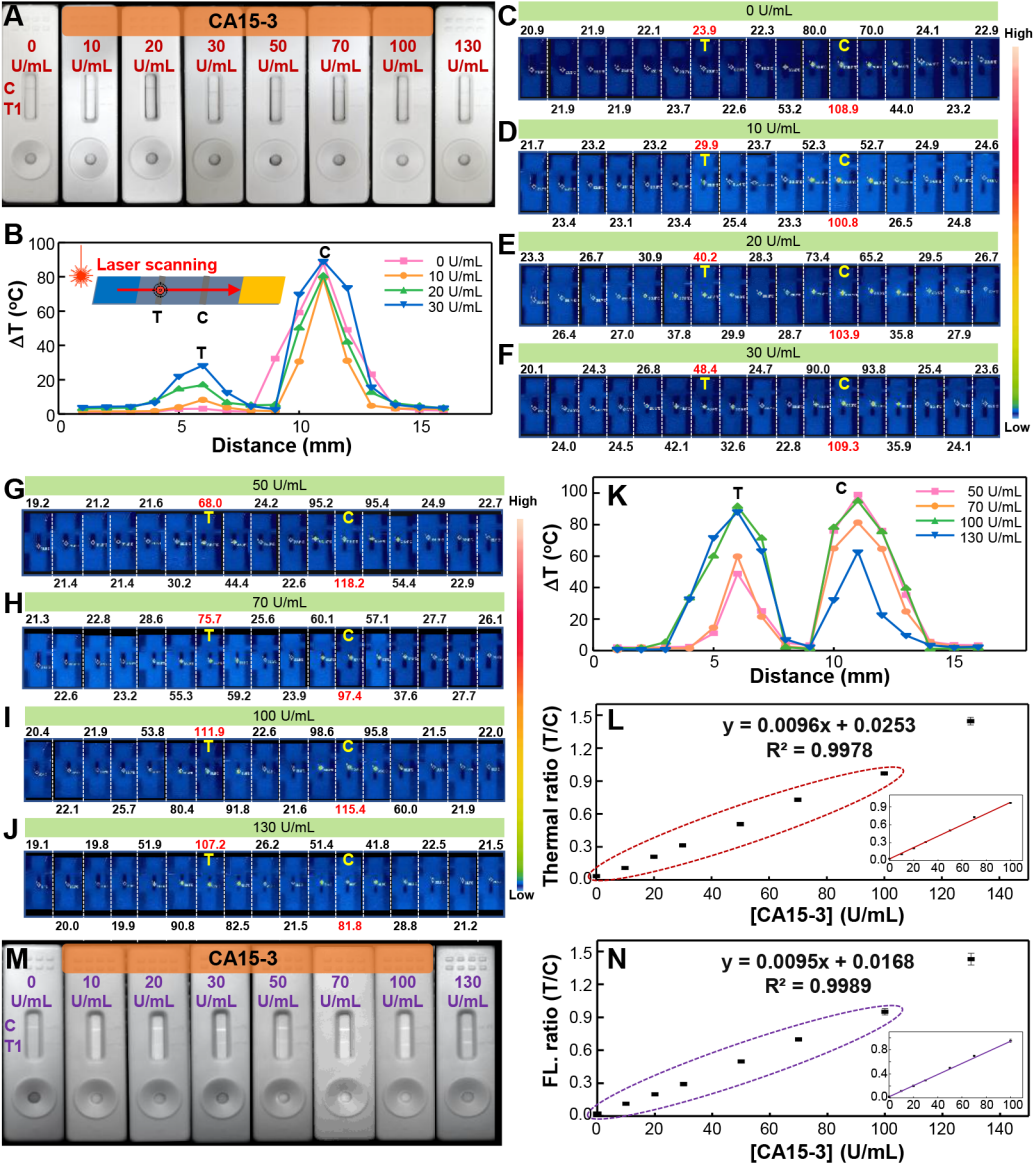
Comparison for the CA15-3 detection sensitivity of thermal contrast and NIR-II fluorescence. (A) Photographs of test strips using CA15-3-conjugated AuNR@Pdots after incubation with samples spanning CA15-3 concentrations from 0 to 130 U/mL. (B) Quantitation of temperature rise by laser irradiation for strips exposed to CA15-3 at 0–30 U/mL. Thermal images of laser-irradiated spots scanned along the sample pad, test and control lines, and absorbent pad for CA15-3 concentrations of (C) 0, (D) 10, (E) 20, (F) 30, (G) 50, (H) 70, (I) 100, and (J) 130 U/mL. The numbers denote the temperatures. (K) Temperature profiles of laser-irradiated spots in test strips for CA15-3 levels of 50–130 U/mL. (M) NIR-II fluorescence images of test strips using CA15-3-conjugated AuNR@Pdots after the reaction with CA15-3-containing samples (0–130 U/mL). Detection linear ranges of CA15-3 were derived from (L) the thermal signal and (N) the NIR-II fluorescence signal.

Because NIR-II Pdots generally exhibit low fluorescence quantum yields (<3%; 1.46% for BDT-TTQ Pdots in this work), their photothermal contribution is expected to play a role in the overall temperature rise under irradiation. We therefore compared the photothermal performances of bare Pdots, AuNRs, and AuNR@Pdots (Figure S3). Upon laser irradiation, temperature increases of 13.7 °C, 40.6 °C, and 50.6 °C, respectively, were observed. These results indicate that the photothermal efficiency of AuNRs is approximately three times higher than that of bare Pdots. Thus, in the AuNR@Pdot nanohybrid, Pdots function primarily as fluorescent reporters, whereas AuNRs serve as the dominant photothermal agents. Based on these results, test line 2 was configured to report only the NIR-II fluorescence signal to simplify probe preparation. Moreover, the invisibility of NIR-II emission to the naked eye prevents interference with rapid CA15-3 screening at test line 1. Test line 2 is designed to diagnose the presence of CEA, a nonspecific serum biomarker that is elevated in many malignancies. As shown in Figure 5, NIR-II Pdots enabled the detection of CEA across a 0–10 ng/mL range, achieving a limit of detection as low as 0.096 ng/mL.

**Figure 5:**
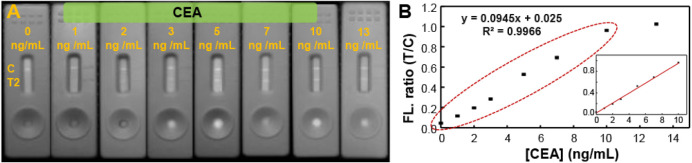
(A) NIR-II fluorescence images of test strips prepared with CEA-labeled Pdots following exposure to analyte solutions containing 0–13 ng/mL of CEA. (B) The corresponding detection range for CEA, with the inset displaying the calibration curve for concentrations between 0 and 10 ng/mL.

### Multiplexed Diagnosis of CA15-3/CEA in LFA

We further implemented a multiplex detection strategy to simultaneously analyze CA15-3 and CEA, enhancing diagnostic accuracy and sensitivity while reducing the assay time and sample consumption. In multiplexing detection, three lines were patterned on the test strip (Scheme 1): a control line, test line 1 (CA15-3), and test line 2 (CEA). Four possible scenarios are anticipated: 1) a negative sample produces no signal on either test line; 2) a CA15-3-positive sample yields a blue–gray band on test line 1 with accompanying NIR-II fluorescence; 3) a CEA-positive sample produces a fluorescence signal on test line 2 in the NIR-II region; and 4) a dual-positive sample develops both test lines with three distinct NIR-II fluorescence bands. The results are displayed in Figure 6 in which minimal cross-interference is observed. This demonstrates the excellent specificity of the probes, enabling the simultaneous detection of multiple targets in complex samples with negligible cross-interference.

**Figure 6:**
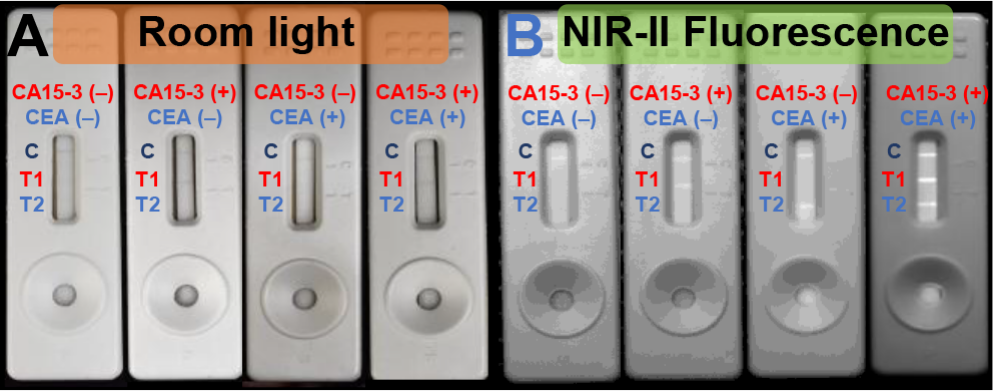
Multiplexing detection for analytes containing CA15-3 and/or CEA. Test strip images under (A) room light and (B) 650 nm flashlight illumination. For positive samples, the concentrations of CA15-3 and CEA were 100 U/mL and 10 ng/mL, respectively.

### Assessment of CEA and CA15-3 Concentrations in Clinical Serum Samples

The analytical capability of the developed LFA, both qualitative and quantitative, was evaluated using serum specimens from healthy donors, as well as patients with breast or lung cancer (Figure 7). In samples from healthy volunteers (upper panel, Figure 7), both test lines exhibited only weak or negligible fluorescence signals, indicating that the concentrations of CA15-3 (test line 1) and CEA (test line 2) were below their respective clinical cutoff values of 31.3 U/mL and 3–5 ng/mL.^31–33^ In contrast, for patients with breast cancer (patients #1, #3, #5, and #6), a distinct test line 1 was clearly visible to the naked eye, enabling rapid on-site screening. When test line 1 appeared negative, the fluorescence intensity of test line 2 served to further assess the potential presence of other malignancies, such as lung cancer (patients #2, #4, and #7). Quantitative analysis of tumor marker levels obtained by the LFA was compared with electrochemiluminescence immunoassay (ECLIA) data from clinical laboratories (Table 1 and Figure 8), revealing a strong consistency between the two methods. It should be noted that certain clinical samples were diluted severalfold to ensure that their measured values remained within the assay’s dynamic range. These results highlight the clinical applicability and diagnostic reliability of the Pdot-based LFA platform.

**Figure 7:**
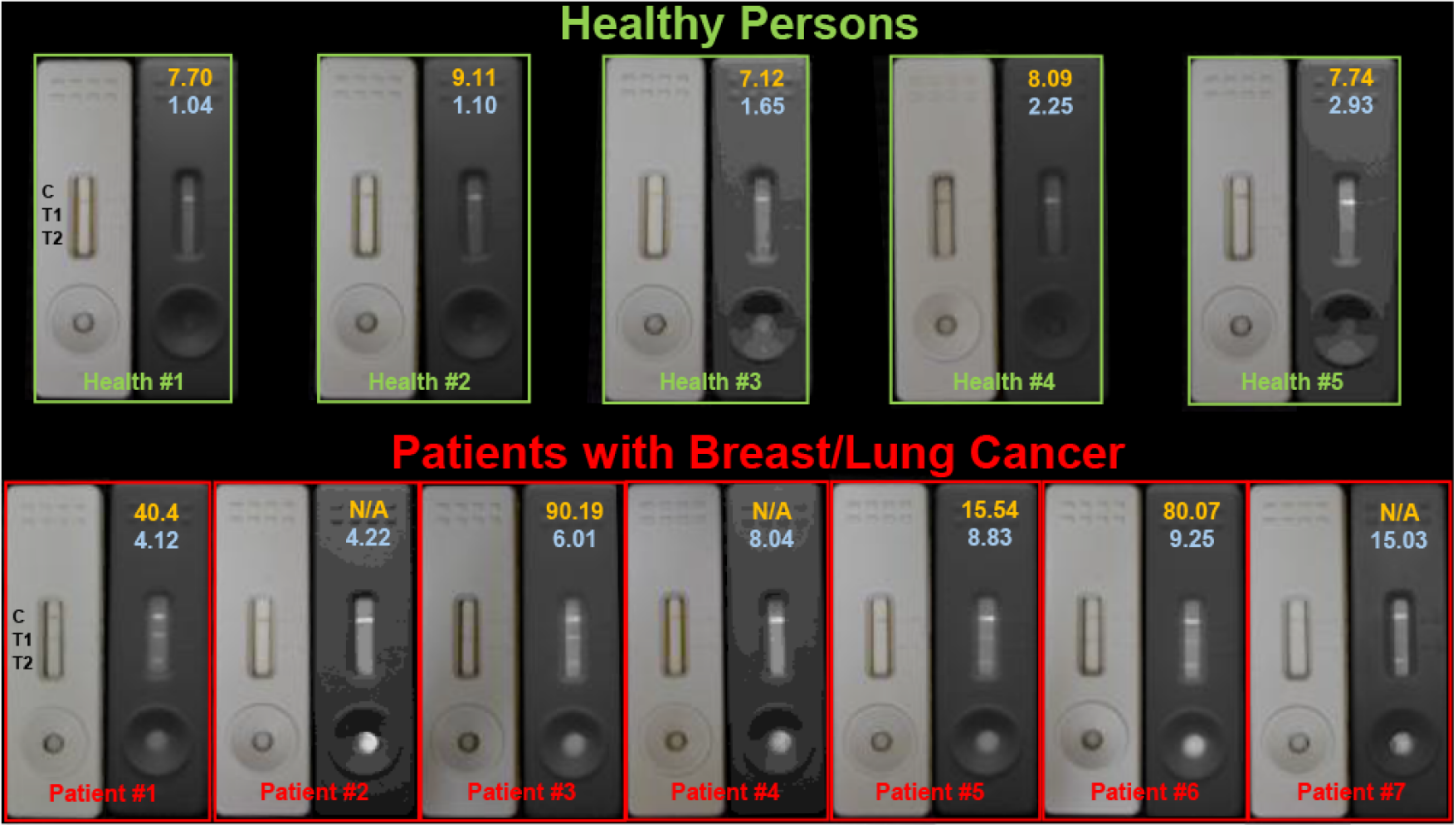
Quantitative evaluation of clinical samples was performed using the Pdot-based LFA, including five samples from healthy volunteers (top) and seven samples from patients diagnosed with breast or lung cancer (bottom panel). In the photographs, the left side displays the test strips under ambient lighting, whereas the right side shows their corresponding NIR-II fluorescence images. The numeric values on each strip represent concentrations measured by electrochemiluminescence immunoassay (ECLIA), with orange numbers indicating CA15-3 levels (U/mL) and blue numbers representing CEA concentrations (ng/mL).

**Table 1:** Comparison of CEA and CA15-3 Levels in Clinical Samples Determined by ECLIA and Present LFA

	[CA15-3]^a^ U/mL	[CEA]^a^ ng/mL	Histology	[CA15-3]^b^ U/mL	[CEA]^c^ ng/mL
Health #1	7.70	1.04		7.59/8.27	1.00
Health #2	9.11	1.10		12.50/10.49	1.17
Health #3	7.12	1.65		6.98/6.36	1.72
Health #4	8.09	2.25		9.38/7.89	2.44
Health #5	7.74	2.93		8.20/7.90	2.92
Patient #1	40.40	4.12	Luminal A	44.48/43.77	4.41
Patient #2	N/A	4.22	Adenocarcinoma	N/A	4.25
Patient #3	90.19	6.01	Luminal A	87.61/90.73	6.06
Patient #4	N/A	8.04	Adenocarcinoma	N/A	8.20
Patient #5	15.54	8.83	Luminal B	16.25/15.70	8.56
Patient #6	80.07	9.25	Luminal A	84.52/81.66	8.77
Patient #7	N/A	15.03	Adenocarcinoma	N/A	15.99

a Clinical samples determined by ECLIA.

b Values determined by test strips using fluorometric (left values) and thermometric (right values) modalities.

c Values determined by test strips using NIR-II fluorescence. N/A: not available.

**Figure 8:**
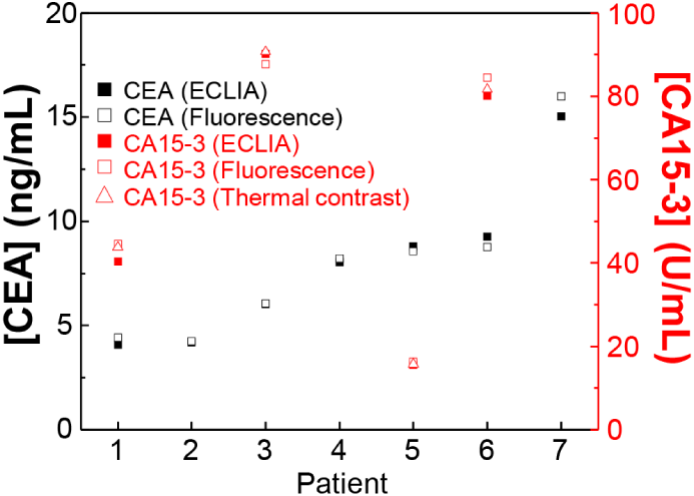
Quantitative determination of CEA (black squares) and CA15-3 (red squares and triangles) in clinical samples containing seven patients with breast cancer (nos. 1, 3, 5, and 6) or lung cancer (nos. 2, 4, and 7) by LFA and ECLIA.

## Conclusions

We developed a multimodal LFA platform that successfully integrates NIR-II fluorescence and photothermal detection for enhanced sensitivity and multiplexed analysis of cancer biomarkers at the point of care. By coupling NIR-II emissive Pdots with Au nanorods, we achieved dual-mode readouts with comparable limits of detection for CA15-3 and high selectivity for CEA. The platform combines the advantages of colorimetric rapid screening, photothermal quantification, and invisible NIR-II fluorescence for simultaneous multianalyte detection without optical interference. Furthermore, clinical validation confirmed excellent agreement between our LFA results and conventional ECLIA assays, which demonstrates its translational potential for accurate and real-time cancer diagnostics. Beyond oncology, this design principle can be broadly extended to other disease biomarkers, marking an important step toward next-generation, high-performance point-of-care testing devices.

## Supplementary Material





## Notes

Additional experimental section of testing processes in real samples (PDF)
Video of testing processes in real samples (MP4)
