# Supplementary material for: Sensitivity Enhancement of Multiplex Lateral Flow Immunoassays by NIR-II Fluorescence and Thermal Contrast
Source: Anal Chem. 2026 Feb 3;98(6):4801–9. doi: 10.1021/acs.analchem.5c06734 (PMC12921665; doi:10.1021/acs.analchem.5c06734)
Supplement: Supplementary file 1 [file ac5c06734_si_001.pdf]

Supporting Information

Sensitivity Enhancement of Multiplex Lateral Flow Immunoassays by NIR-II Fluorescence and Thermal Contrast

Yi-Chi Luo,[a†] Yung-Chun Hsieh,[b†] Chun-Yang Huang,[a] Yu-Jun Liu,[a] Hsin-Ting Huang,[a] Yan-Chang Chen,[a] Tsung-Yuan Wang,[c] Chong-You Chen,[c] and Yang-Hsiang Chan*[ade]*

[†]Authors contributed equally to this work

[a]Department of Applied Chemistry, National Yang Ming Chiao Tung University, Hsinchu, Taiwan 30010

[b]Department of Surgery, National Taiwan University Hospital, Hsinchu Branch, Hsinchu, Taiwan 30010

[c]Department of Chemistry, National Taiwan Normal University, Taipei, Taiwan 11677

[d]Center for Emergent Functional Matter Science, National Yang Ming Chiao Tung University, Hsinchu, Taiwan 30010

[e]Department of Medicinal and Applied Chemistry, Kaohsiung Medical University, Kaohsiung, Taiwan

E-mail: Chong-You Chen (jasoncyc@ntnu.edu.tw), Yang-Hsiang Chan (yhchan@nycu.edu.tw)

Supporting Information

Contents

Experimental Procedures S3
Materials. S3
Synthetic Procedures of Polymers S4
Characterization and Preparation of LFA S5
Figure S1 .... S5
Figure S2 .... S8
Figure S3 .... S9
Table S1 .... S9
Table S2 .... S9
Table S3 .... S10
Table S4 .... S10
Figure S4 .... S11
Figure S5 .... S11
Figure S6 .... S12
Figure S7 .... S12
NMR/HRMS data S13
References: S16

Supporting Information

Experimental Procedures

<u>*Materials.*</u>
The chemicals except for bio-related reagents were obtained from Sigma-Aldrich, Acros, TCI, and Thermo Fisher. Highly pure water (18.2 MΩ•cm) was used throughout our experiments. Polystyrene graft ethylene oxide functionalized with carboxyl acid group (PS-PEG-COOH, M_n = 6500, PDI=1.3) was obtained from Polymer Source (Quebec, Canada) without further purification. Semiconducting polymers **PFCN**,[1] and **BDT-TTQ**[2] were synthesized according to the reported literatures. CYFRA21-1 and CEA antibodies and antigens were acquired from Fitzgerald (MA, USA, now Biosynth). Mouse anti-human CA15-3 antibodies (Cat. No.: 136551 and 136549) were engineered onto the test line and the Pdot surface, respectively. Mouse anti-human CEA (catalog number: 10-7882 and 10-7883) were conjugated onto the probe and the test line, respectively. AffiniPure goat antimouse IgG (H+L) secondary antibodies (AB_2338447) were acquired from Jackson ImmunoResearch (West Grove, PA) for further fabrication on the control line. CA15-3 antigen (C0050-27) and CEA antigen (30-AC26) were diluted into the desired concentrations by PBS buffer upon use. Nitrocellulose membranes (5 μm, CNPC), sample pads (GFB-R4), conjugate release matrices (PT-R5), and absorbent pads (AP080) were obtained from mdi Membrane Technologies and then cut into desired the dimension to assemble to test strips, and then inserted into 4 mm plastic cassettes. Olympus OM-D E-M10 Mark III camera equipped with an 830-nm long-pass filter was ordered from Life Pixel Infrared (WA, USA).

Supporting Information

Scheme S1. Synthetic routes for the **BDT-TTQ** conjugated polymer.

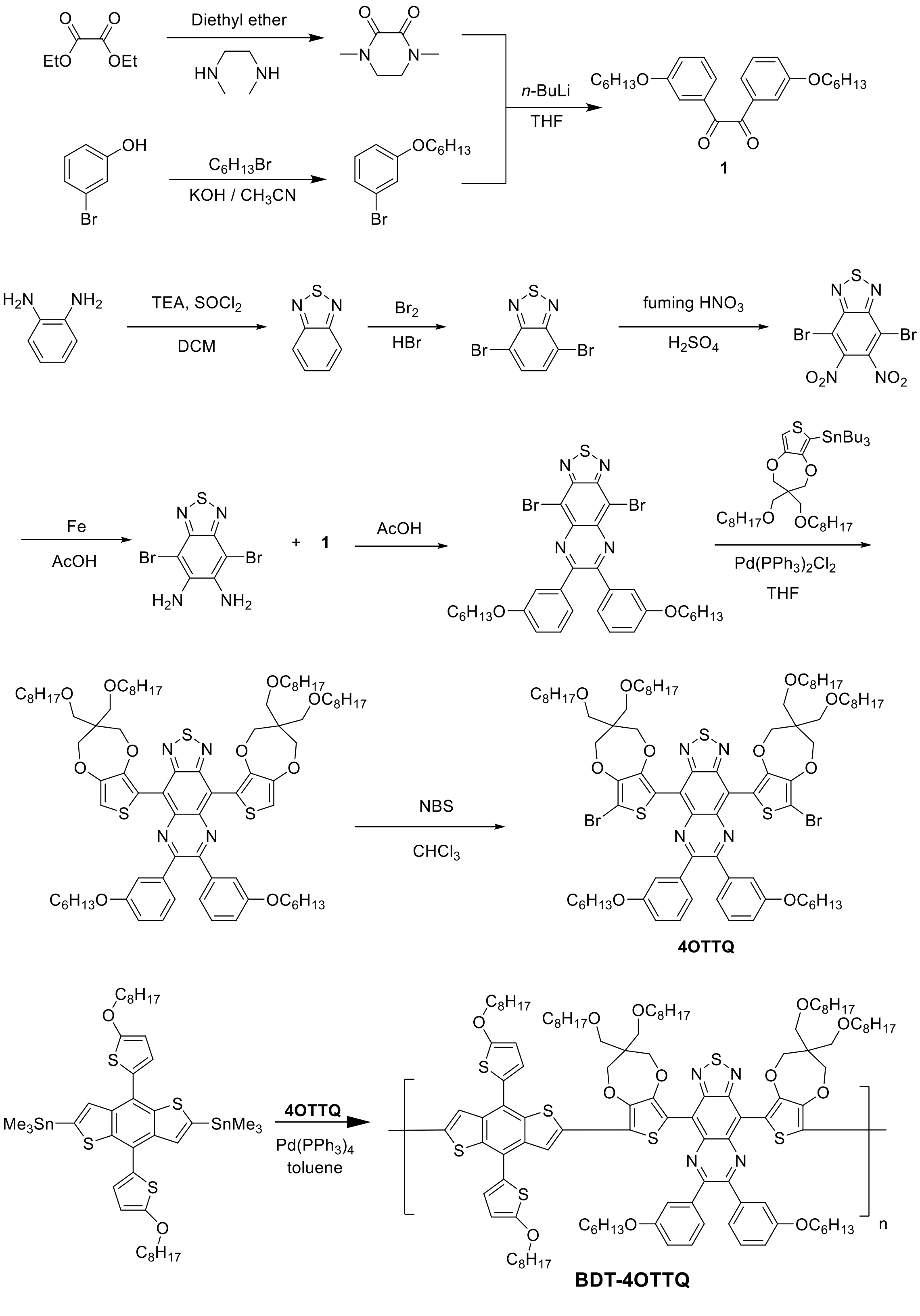

Supporting Information

Optimization of Fluorescence Enhancement of AuNR@Pdots and Characterization of AuNR@Pdot Probes

Since Fluorescence quenching via energy or electron transfer and plasmon-induced emission enhancement are competing processes that are strongly governed by the spatial separation between fluorophores and metal nanostructures. When Pdots are positioned in close proximity to AuNRs, nonradiative decay pathways dominate and thus lead to fluorescence suppression. Consequently, controlling the effective Pdot shell thickness on the AuNR surface is critical for maximizing emission enhancement. To address this point, we conducted systematic optimization experiments and determined that a Pdot-to-AuNR ratio of 0.3 yields the maximum fluorescence amplification as shown below, reaching approximately a 1.5-fold increase.

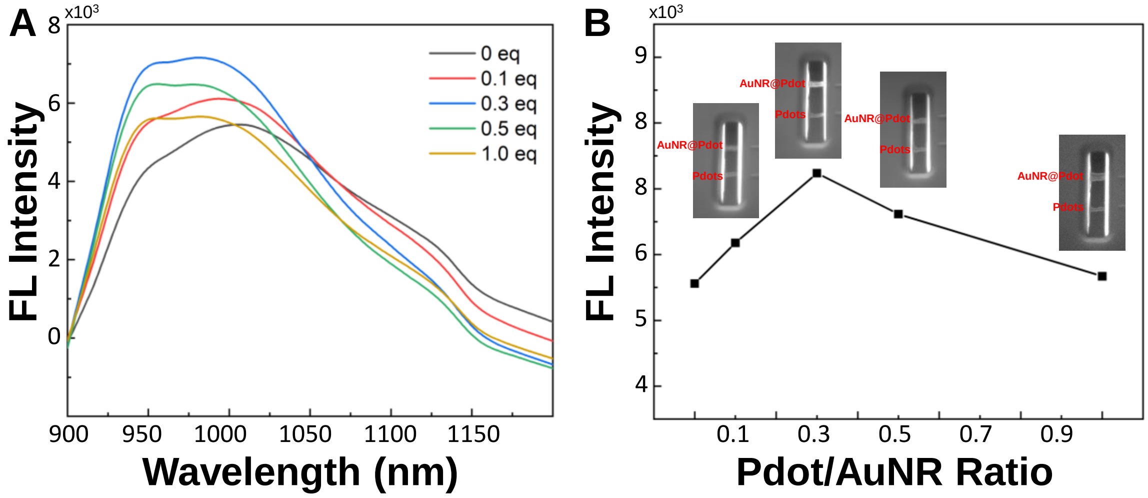

Figure S1. (A) Fluorescence spectra of bare Pdots (0 eq.) and AuNR@Pdots at different Pdot-to-AuNR ratios (0.1-1 eq). (B) Their corresponding lines fabricated on test strips (upper lines) as compared to bare Pdots (bottom lines).

A Dynamica Limited DB20S UV-visible spectrometer was used for UV/Visible spectra. A Malvern Zetasizer Nano S measured the average hydrodynamic radius via DLS. Images of the Pdots were captured using a Hitachi HT7700 transmission electron microscope (TEM) at 100 kV. For TEM, a 5 μL drop of a diluted Pdot solution was placed on a carbon-coated grid. The grid was dried in a cabinet for 24 hours and then heated in an 80 °C oven for another 24 h to remove all water. Finally, fluorescence spectra were measured with an Edinburgh Instruments Ltd. FS5 spectrofluorometer.

Supporting Information

Preparation of Test Strip by Using Pdot-Based Probes

Scheme 1 illustrates the assembly of the different pads on the test strip. First, we cut the sample pad and absorbent pad into 6 x 3 mm^2. Using an automated lateral flow reagent dispenser, the 3 mm wide nitrocellulose membrane was fabricated. For single-analyte detection, a test line was composed of either CEA (10-7883, 0.2 mg/mL) or CA15-3 (136551, 0.3 mg/mL) antigens, while a control line contained IgG secondary antibodies (0.8 mg/mL). For multiplexed detection, two separate test lines were loaded with CEA and CA15-3. After fabrication, the test strip was placed under vacuum for 3 min to dry the water. The dried test strip was then assembled by integrating the absorbent and sample pads with the nitrocellulose membrane. Finally, the complete strip was inserted into a 4 mm plastic cassette.

Detection of CA15-3/CEA by AuNR@Pdot-Based LFA

To prepare the running buffer, a mixture of various substances was combined. Specifically, 15 μL of 5% (w/w) Triton X-100, 5 μL of 1% (w/w) glycerol, and 5 μL of 5% (w/w) polyethylene glycol 3000 were mixed together. Additionally, 8 μL of Pdot-based probes, X μL of the target solution containing CEA and/or CA15-3, and (67-X) μL of 20 mM HEPES buffer were added to the mixture. For the calibration samples used to establish the calibration curves, CA15-3/CEA were spiked into the running buffer. Volumes ranging from 0 to 20 μL of the spiked solution were added to the running buffer, resulting in a final volume of 0.1 mL. For real samples from patients, 70 μL of 20 mM HEPES, 10 μL of Pdot-based probes, 10 μL of 5% Triton X-100, 10 μL of 5% PEG, 5 μL of ethylene glycol, and 5 μL of serum samples from patients. For real samples from healthy volunteers, 5-10 μL of whole blood samples were used. After that, the test strips were left undisturbed for a duration of 15 min until the results became visible. The fluorescence images of the test

strips can be captured using an Olympus OM-D E-M10 Mark III digital camera or a Ninox 640 II InGaAs NIR-II camera and the data were analyzed by ImageJ software.

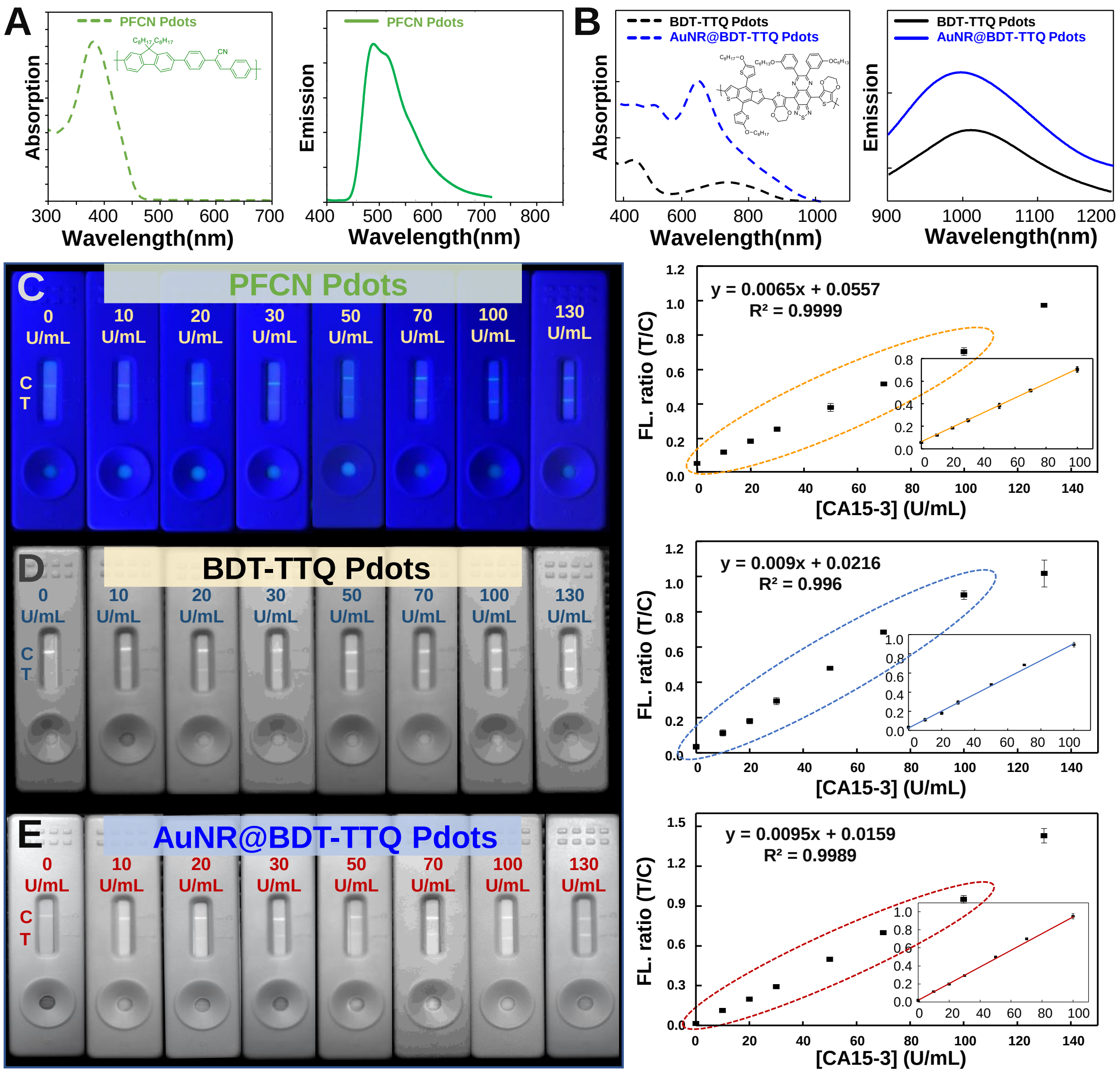

Figure S2. Sensitivity comparison of visibly emissive PFCN Pdots, NIR-II fluorescent BDT-TTQ Pdots, and AuNR@BDT-TTQ hybrids. (A) Absorption (left) and emission (right) spectra of PFCN Pdots. (B) Absorption (left) and emission (right) spectra of BDT-TTQ and AuNR@BDT-TTQ Pdots. Fluorescent photographs under UV excitation after reacting with analytes containing CA15-3 of 0-130 U/mL by using (C) PFCN Pdots, (D) BDT-TTQ Pdots, and (E) AuNR@BDT-TTQ Pdots as probes. Their corresponding dynamic ranges of CA15-3 were shown on the right panels.

Supporting Information

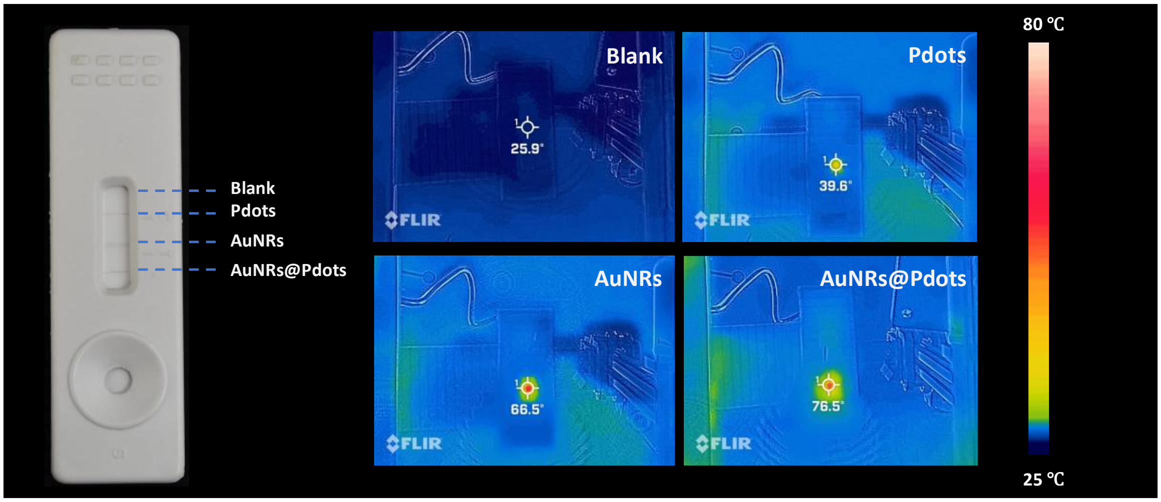

Figure S3. Comparison of thermal contrast of different probes on the test strip, showing that AuNR@Pdots have the best performance.

Table S1. Comparison of CEA detection performance of this NIR-II fluorometric LFA with reported fluorescent LFAs.

CEA	Probe	LOD (ng/mL)	Linear Range (ng/mL)	Multi-modal	Reference
NIR-II emission	Pdots (λ_{em}=1010 nm)	0.096	0-10	No	This Work
Visible emission	AuNP@FITC (λ_{em}=520 nm)	0.1	5-80	Yes	[3]
NIR-II emission	RENPs (λ_{em}=1064 nm)	0.37	1-320	No	[4]
Visible emission	Quantum Dots	1	1-100	No	[5]
Visible emission	Quantum Dots	0.35	2.8-680	No	[6]
NIR-II emission	Polystyrene Beads	0.768	5-300	No	[7]

Table S2. Comparison of CA15-3 detection performance of this multimodal LFA with reported LFAs.

CA15-3	Probe	LOD (U/mL)	Linear Range (U/mL)	Multi-modal	Reference
Thermometry<br>NIR-II emission	AuNR@Pdots (λ_{em}=1010 nm)	0.40<br>0.42	0-100	Yes	This Work
NIR-II emission	Polystyrene Beads	1.192	8.2-410	No	[7]
Fluorescence	PLGA@MNP	0.09	0-200	Yes	[8]

Table S3. Comparison of readout methods and detection strategies in LFAs.

Readout method	Label medium	Signal	Detector
Colorimetric	Colloidal nanoparticles	Optical density / color contrast	Charge-coupled device (CCD) camera
Fluorescence	Monodisperse fluorescent nano-materials	Fluorescence emission intensity	Charge-coupled device
Magnetic	Paramagnetic latex or superparamagnetic Fe_2O_3-based nanoparticles	Magnetic field enhancement	Inductive magnetic sensor
Photothermal	Plasmonic metal-based nanoparticles	Photothermal temperature rise	Infrared (IR) thermal camera
Electrochemical	Conducting nanoparticles	Electrical potential or current change	Potentiostat / galvanometer
SERS	Surface-engineered metal nanoparticles	Raman spectral shift	Raman detector

Table S4. Comparison of multi-modal LFA versus traditional single-mode LFA.

Parameters	Dual/Multi mode LFA	Single mode LFA	Comments/Analysis
Sensitivity	Enhanced sensitivity enabled by integration of multiple readout modes	Dependent on a single signal transduction pathway	Multimode LFAs achieve improved sensitivity by combining complementary signaling mechanisms, thereby overcoming intrinsic limitations of single-mode formats
Complexity and Cost	Increased assay complexity and fabrication cost due to multimodal integration	Simpler assay architecture with lower production cost	Although multimode LFAs are more complex and costly, the performance gains in sensitivity and functionality may justify their use in advanced applications
Assay Speed	Potentially longer assay time resulting from increased system complexity	Typically faster owing to streamlined design	Single-mode LFAs are advantageous for rapid testing, whereas multimode LFAs may trade speed for improved analytical performance
Accessibility	May require specialized instrumentation for certain readout modes	Highly accessible, particularly in resource-limited settings	Single-mode LFAs offer broader accessibility, while multimode LFAs are better suited for settings where additional instrumentation is available
Versatility	Highly versatile, supporting multiple readout strategies for diverse applications	Restricted to a specific readout modality	The multimode approach enables broader applicability across analytical scenarios, whereas single-mode LFAs are more application-specific
Typical Applications	Clinical diagnostics, environmental analysis, and food safety monitoring	Targeted applications dictated by the selected readout mode	Multimode LFAs provide comprehensive solutions across fields, while single-mode LFAs are optimized for specific use cases

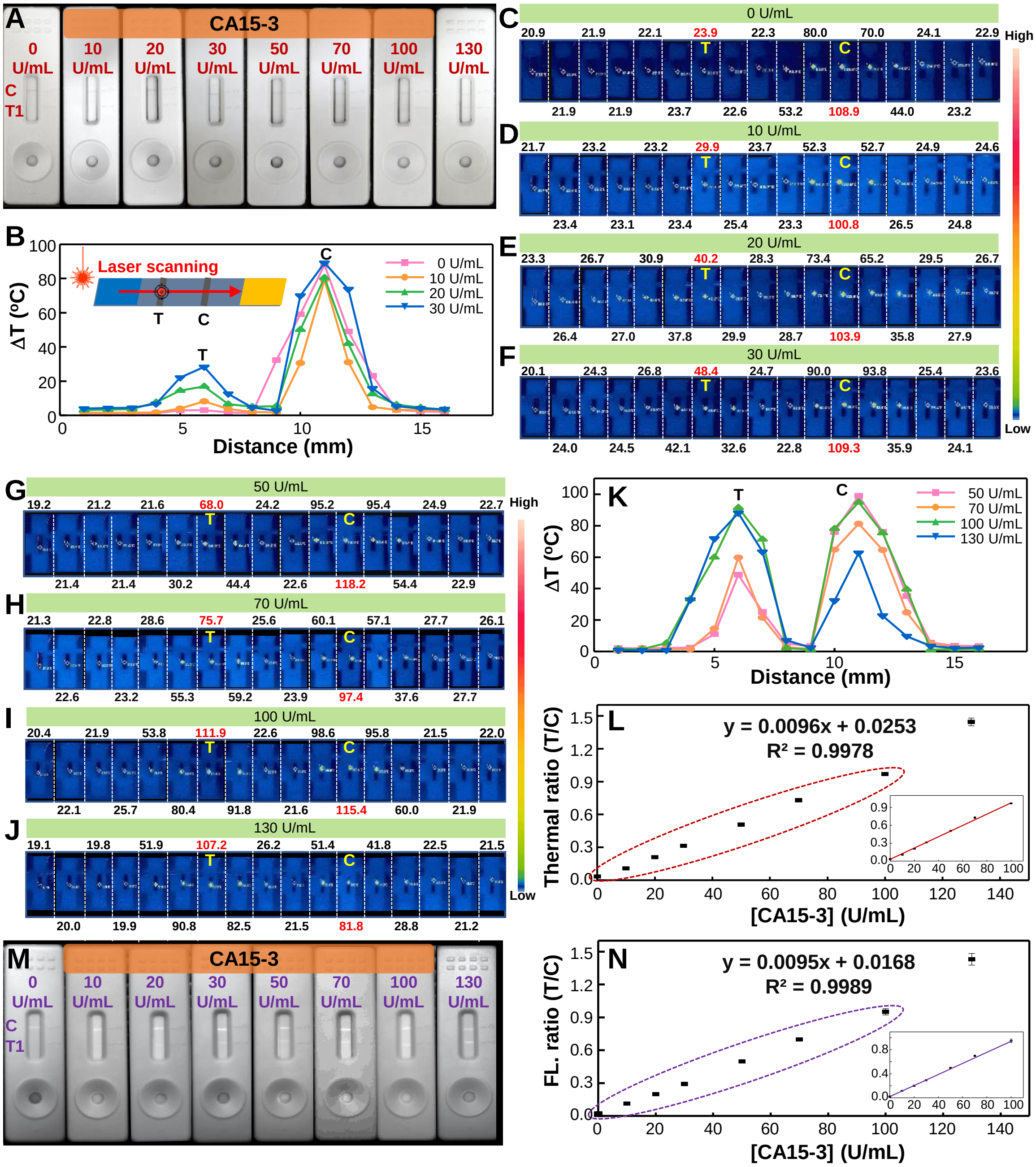

Figure S4. High-resolution image of Figure 4.

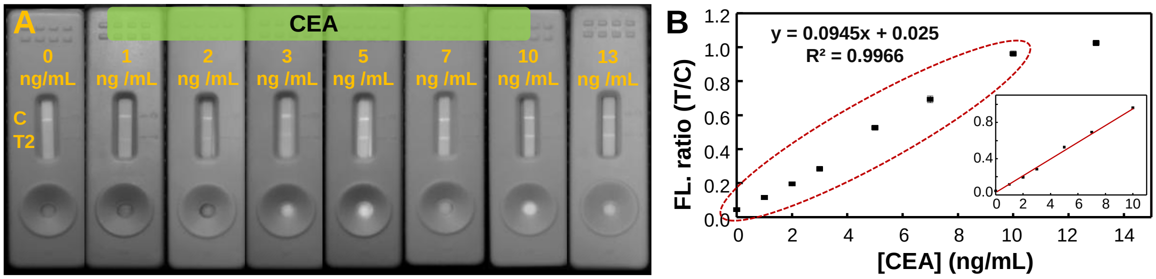

Figure S5. High-resolution image of Figure 5.

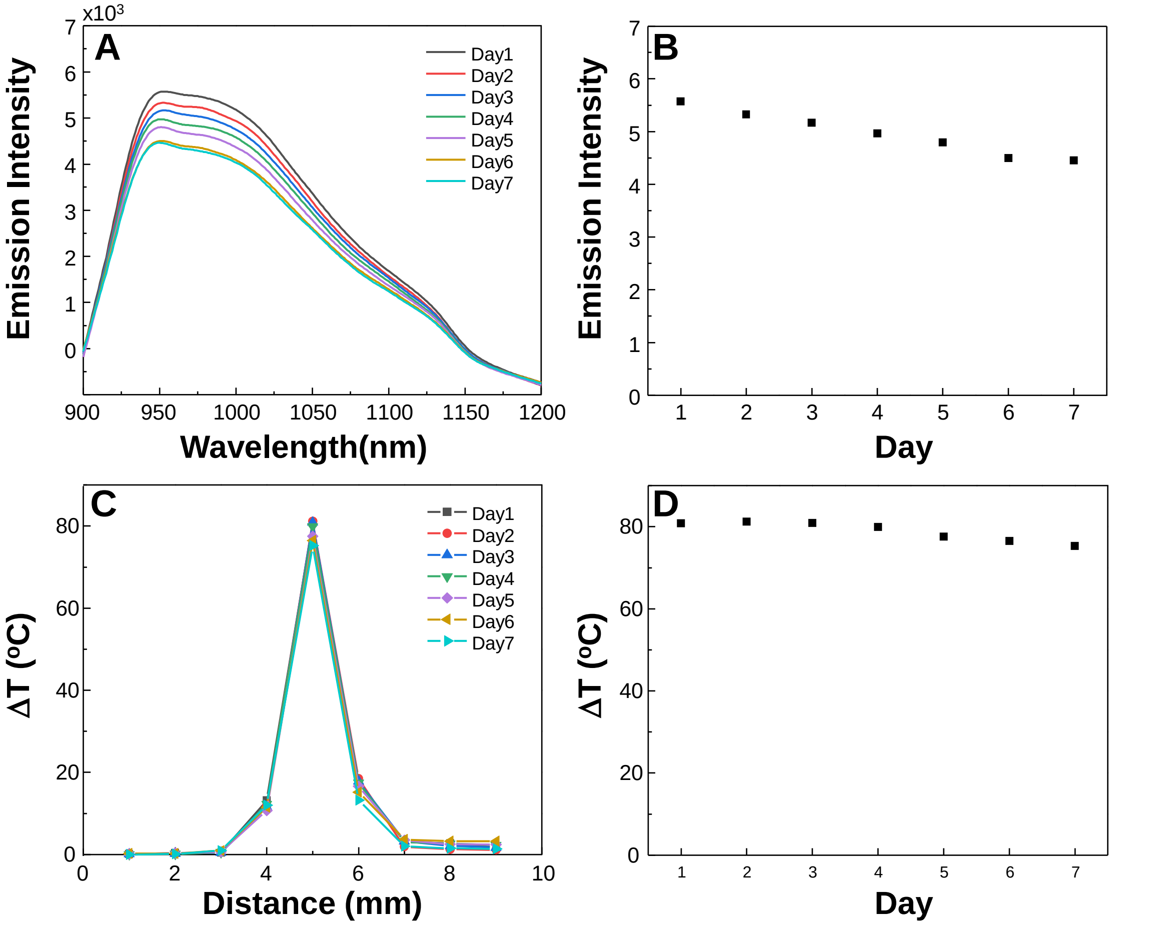

Figure S6. The evaluation of storage stability of AuNR@Pdots at room temperature in the dark. (A) NIR-II fluorescence spectra of AuNR@Pdots stored at different days and (B) their corresponding emission intensities. (C) Photothermal efficiency of AuNR@Pdots fabricated on the test strips at different days and (D) their corresponding thermal contrast upon laser irradiation.

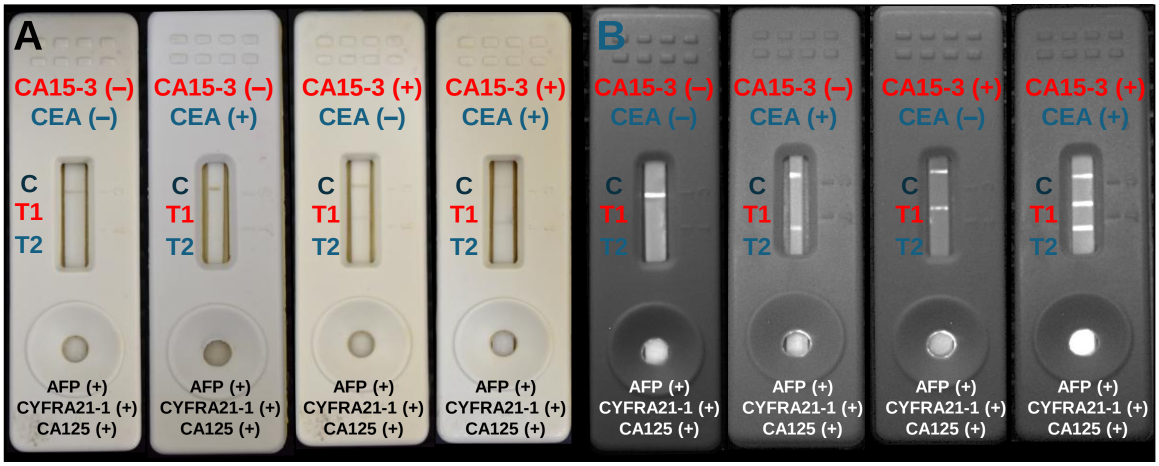

Figure S7. Multiplexing detection for CA15-3 and CEA. Test strip images under (A) room light and (B) under 650-nm flashlight illumination. For positive samples, the concentrations of CA15-3, CEA, AFP, CYFRA21-1, and CA125 were 100 U/mL, 10 ng/mL, 10 ng/mL, 10 ng/mL, and 36 ng/mL, respectively.

Supporting Information

1,4-dimethylpiperazine-2,3-dione ^{1}H NMR (400 MHz, $CDCl_3$): δ = 3.55 (s, 4H), 3.08 (s, 6H). HRMS (FD, $[M]^{+}$) for $C_6H_{10}N_2O_2$ calcd.: 143.08150, found: 143.08169

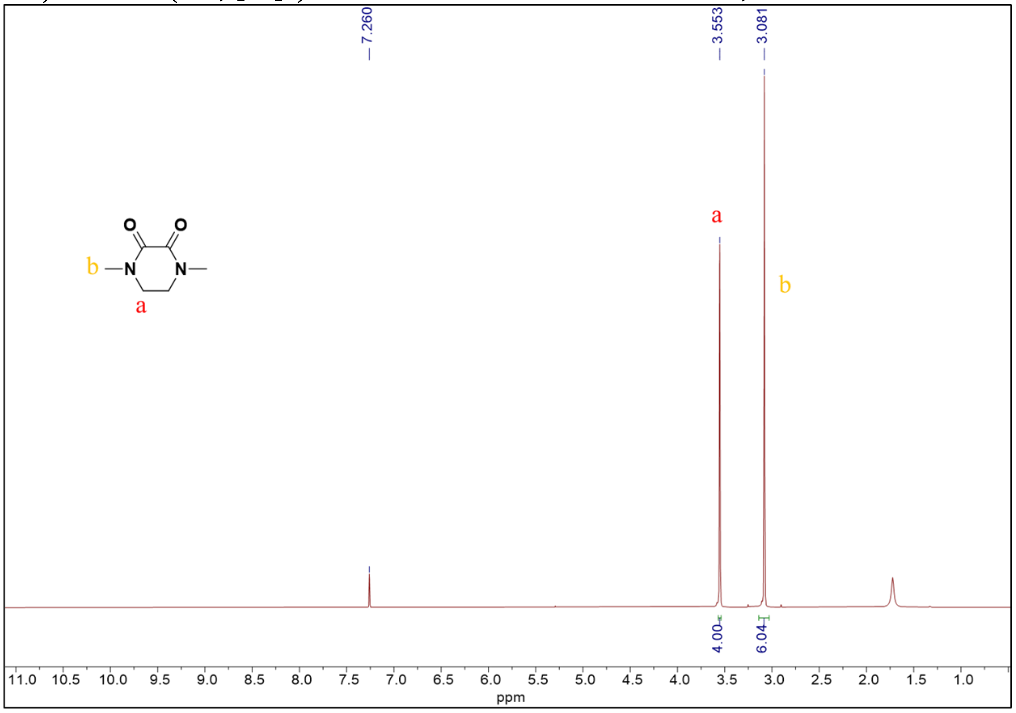

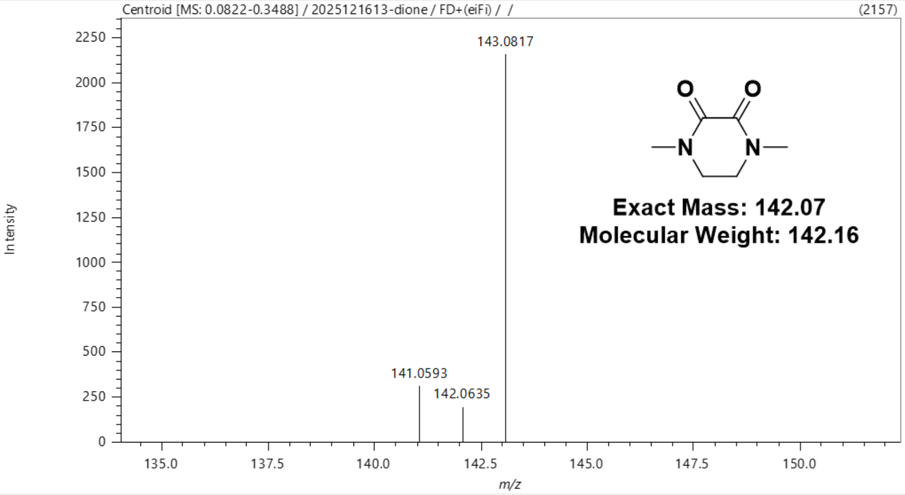

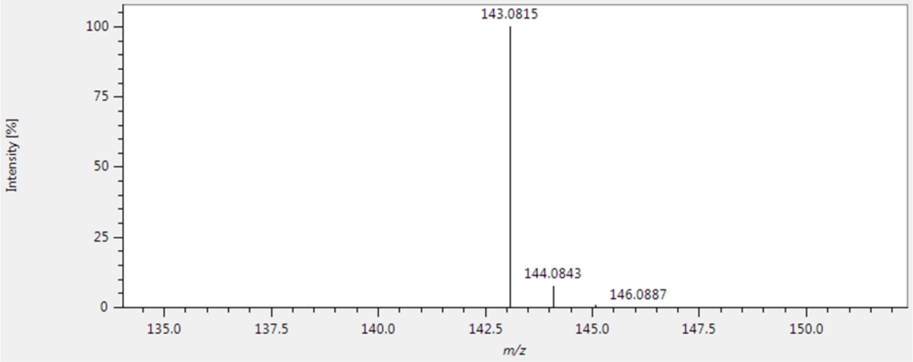

	Mass	Intensity	Formula	Calculated Mass	Mass Difference [mDa]	Mass Difference [ppm]	DBE
▸	143.08169	2156.70	C6 H11 N2 O2	143.08150	0.18	1.28	2.5

1-bromo-3-hexyloxybenzene ^{1}H NMR (300 MHz, $CDCl_3$): δ = 7.10 (t, J = 8.4 Hz, 1H), 7.05~7.02 (m, 2H), 6.81~6.78 (m,1H), 3.90 (t, J = 6.4 Hz, 2H), 1.75 (m, J = 6.8 Hz, 2H), 1.46~1.39 (m, 2H), 1.36~1.26 (m, 4H), 0.89 (t, J = 6.8 Hz, 3H).^{13}C NMR (75 MHz, CDCl3): δ = 159.93, 130.40, 123.47, 122.73, 117.69, 113.49, 68.69, 31.51, 29.07, 25.63, 22.56, 13.98. HRMS (FD, $[M]^+$) for $C_{12}H_{17}BrO$ calcd.: 256.04573, found: 256.04611

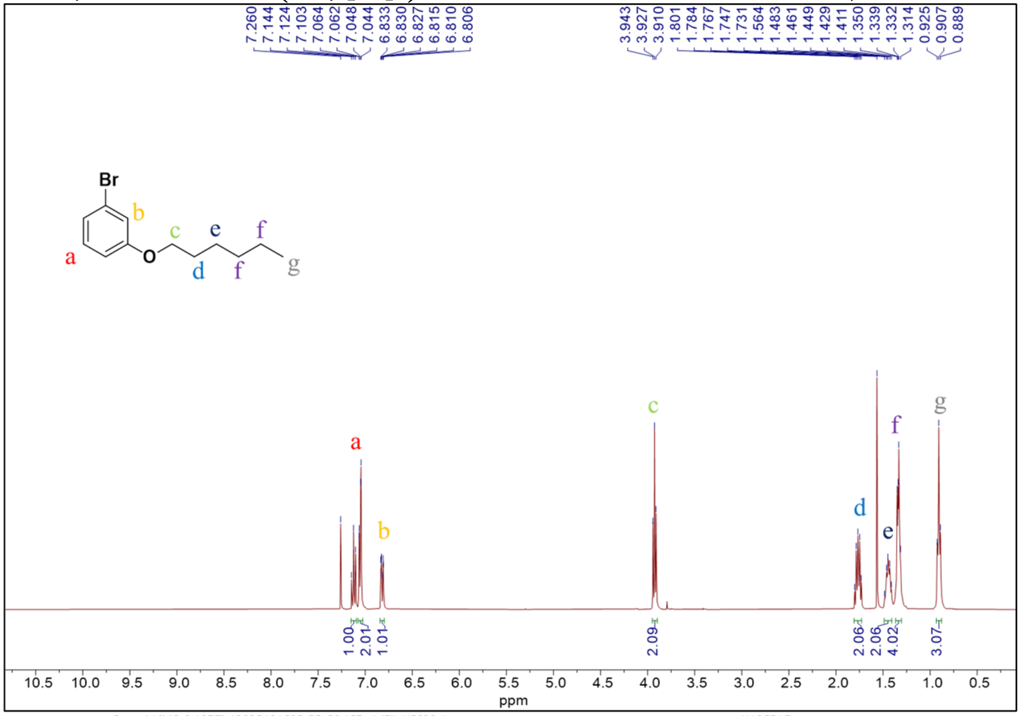

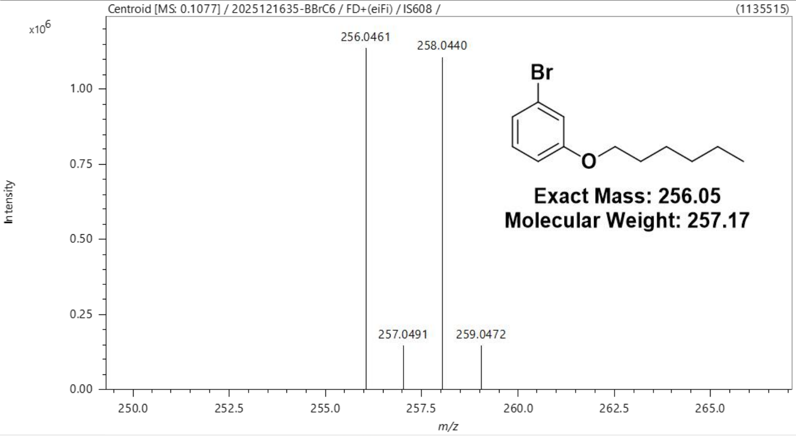

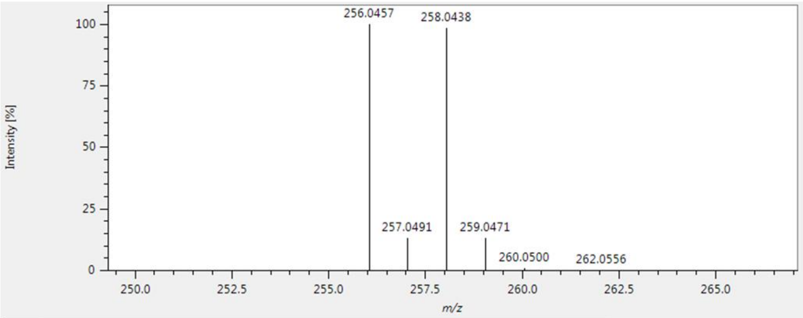

	Mass	Intensity	Formula	Calculated Mass	Mass Difference [mDa]	Mass Difference [ppm]	DBE
▸	256.04611	1135515.00	C12 H17 O Br	256.04573	0.38	1.50	4.0

1,2-Bis(3-hexyloxyphenyl)ethane-1,2-dione, compound 1 ^{1}H NMR (300 MHz, $CDCl_3$): δ = 7.49 (m, 2H), 7.43 (m, 2H), 7.35 (t, J = 10.4 Hz, 2H), 7.18~7.15 (m, 2H), 3.98 (t, J = 6.4 Hz, 4H), 1.77 (m, 4H), 1.47~1.40 (m, 4H), 1.34~1.29 (m, 8H), 0.88 (t, J = 7.2 Hz, 6H). ^{13}C NMR (75 MHz, $CDCl_3$): δ = 194.52, 159.62, 134.21, 129.95,122.91, 122.19, 113.62, 68.35, 31.51, 29.05, 25.63, 22.55, 13.98. HRMS (FD, $[M]^+$) for $C_{26}H_{34}O_4$ calcd.: 410.24516, found: 410.24522

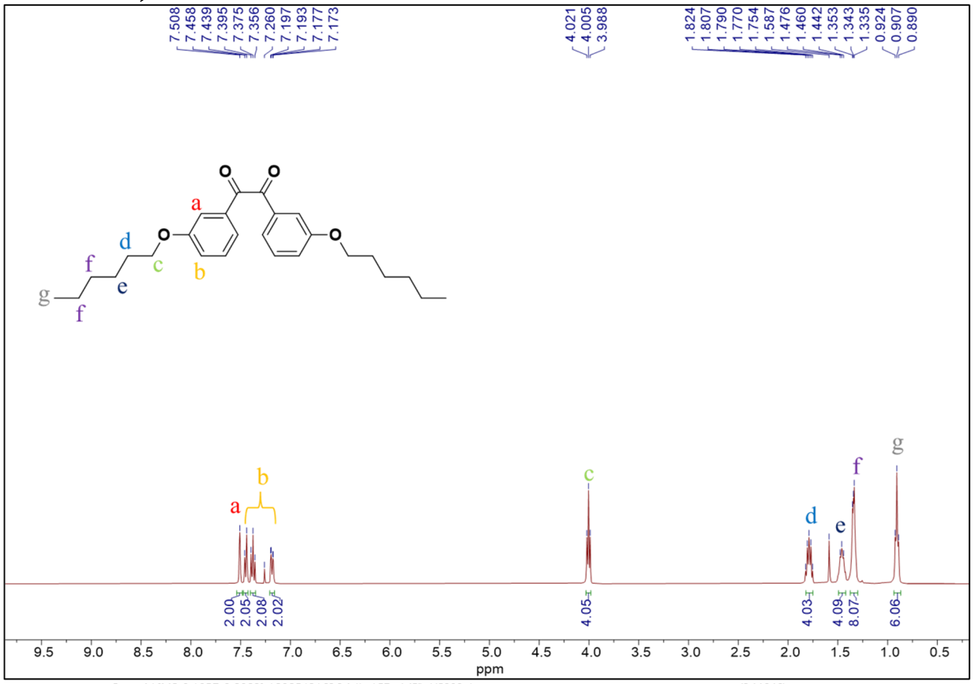

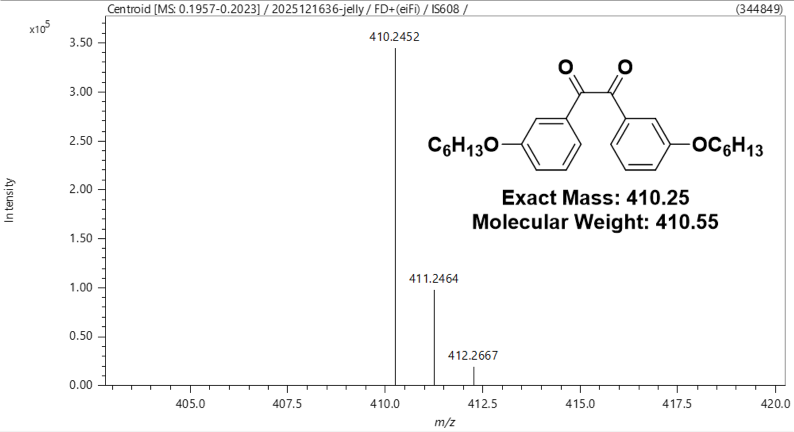

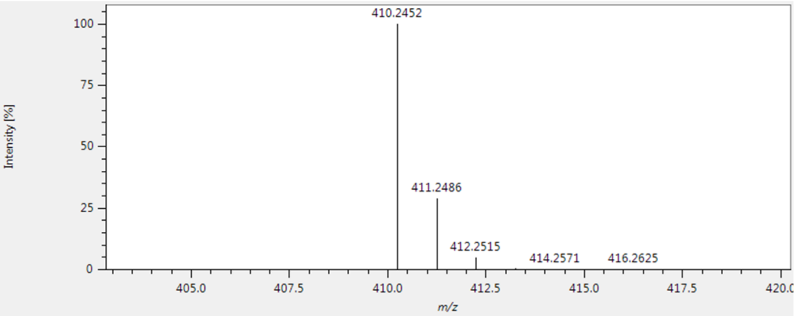

	Mass	Intensity	Formula	Calculated Mass	Mass Difference [mDa]	Mass Difference [ppm]	DBE
▶	410.24522	344848.87	C26 H34 O4	410.24516	0.06	0.14	10.0

References:

[1] Y.-C. Chen, Y.-H. Syu, J.-Y. Huang, C.-Y. Lin, Y.-H. Chan, *Chem. Commun.* **2023**, 59, 9968.

[2] Y.-C. Tsai, Y.-C. Chen, H.-F. Lu, K.-M. Chan, S.-L. Lin, P.-X. Lin, R. Rotomskis, S. Steponkiene, T.-K. Wu, M.-H. Chan, J.-a. A. Ho, Y.-F. Huang, C.-P. Hsu, Y.-H. Chan, *J. Am. Chem. Soc.* **2025**, 147, 21940.

[3] J. Wang, F. Cao, S. He, Y. Xia, X. Liu, W. Jiang, Y. Yu, H. Zhang, W. Chen, *Talanta* **2018**, 176, 444.

[4] Z. Song, Y. Suo, S. Duan, S. Zhang, L. Liu, B. Chen, Z. Cheng, *Biosens. Bioelectron.* **2023**, 224, 115063.

[5] K. Xiao, K. Wang, W. Qin, Y. Hou, W. Lu, H. Xu, Y. Wo, D. Cui, *Talanta* **2017**, 164, 463.

[6] Z. Chen, R. Liang, X. Guo, J. Liang, Q. Deng, M. Li, T. An, T. Liu, Y. Wu, *Biosens. Bioelectron.* **2017**, 91, 60.

[7] K. Deng, Z.-L. Yu, X. Hu, J. Liu, X. Hong, G. G. L. Zi, Z. Zhang, Z.-Q. Tian, *Mikrochim. Acta* **2023**, 190, 462.

[8] B. Zhang, W. Ma, F. Li, W. Gao, Q. Zhao, W. Peng, J. Piao, X. Wu, H. Wang, X. Gong, J. Chang, *Nanoscale* **2017**, 9, 18711.
